# Cell‐Based Identification of New IDO1 Modulator Chemotypes

**DOI:** 10.1002/anie.202016004

**Published:** 2021-03-24

**Authors:** Elisabeth Hennes, Philipp Lampe, Lara Dötsch, Nora Bruning, Lisa‐Marie Pulvermacher, Sonja Sievers, Slava Ziegler, Herbert Waldmann

**Affiliations:** ^1^ Department of Chemical Biology Max Planck Institute of Molecular Physiology Otto-Hahn-Str. 11 44227 Dortmund Germany; ^2^ Department of Chemical Biology Technical University of Dortmund Otto-Hahn-Strasse 6 44227 Dortmund Germany; ^3^ Compound Management and Screening Center Otto-Hahn-Str.11 44227 Dortmund Germany

**Keywords:** cancer, fluorescence, heme proteins, high throughput screening, immunology

## Abstract

The immunoregulatory enzyme indoleamine‐2,3‐dioxygenase (IDO1) strengthens cancer immune escape, and inhibition of IDO1 by means of new chemotypes and mechanisms of action is considered a promising opportunity for IDO1 inhibitor discovery. IDO1 is a cofactor‐binding, redox‐sensitive protein, which calls for monitoring of IDO1 activity in its native cellular environment. We developed a new, robust fluorescence‐based assay amenable to high throughput, which detects kynurenine in cells. Screening of a ca. 150 000‐member compound library discovered unprecedented, potent IDO1 modulators with different mechanisms of action, including direct IDO1 inhibitors, regulators of IDO1 expression, and inhibitors of heme synthesis. Three IDO1‐modulator chemotypes were identified that bind to apo‐IDO1 and compete with the heme cofactor. Our new cell‐based technology opens up novel opportunities for medicinal chemistry programs in immuno‐oncology.

## Graphical abstract

Heme‐competitive indoleamine‐2,3‐dioxygenase (IDO1) small‐molecule modulators are under‐represented as heme displacement is delayed in in vitro assays. This limitation can be overcome by cell‐based screening as employed in this study, which revealed novel chemotypes that potently inhibit IDO1 activity and kynurenine production by competing with heme.

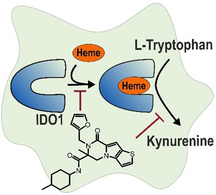

Cancer cells have evolved mechanisms to evade immune cell‐mediated elimination and modulation of these processes is a promising approach in anti‐cancer drug discovery.^[1]^ Indoleamine‐2,3‐dioxygenase 1 (IDO1) is an immunoregulatory enzyme that is induced by pro‐inflammatory cytokines, such as interferon gamma (IFNγ).^[2]^ In various cancers, IDO1 expression is associated with poor prognosis.^[3]^ IDO1 is a heme‐containing protein and catalyzes the conversion of l‐tryptophan (Trp) to N‐formylkynurenine (NFK), which is further degraded to kynurenine (Kyn).^[4]^ IDO1‐mediated depletion of Trp reduces effector T‐cell proliferation, and accumulation of Kyn promotes regulatory T‐cell differentiation, thus reducing anti‐tumor immunity and supporting tumor progression.^[5]^ Hence, inhibition of IDO1 may restore immune‐mediated cancer cell elimination, and various chemotypes were identified as IDO1 inhibitors by means of target‐based approaches.^[6]^ Unfortunately, exploration of IDO1 inhibitors for the treatment of unresectable or metastatic melanoma has had limited success. The IDO1 inhibitor epacadostat in combination with pembrozilumab, an antibody that targets programmed cell death protein 1 (PD‐1), did not outperform treatment with pembrozilumab alone. However, neither tumoral IDO1 expression of selected patients nor Kyn levels as a biomarker for IDO1 were evaluated.^[7]^ Therefore, inhibition of IDO1 by means of new inhibitor chemotypes remains a promising opportunity to enhance immune cell‐mediated elimination of cancer cells. In this context, exploration of alternative binding sites of IDO1, like allosteric inhibition or targeting apo‐IDO1 may be of particular interest.[^7^, ^8^]

Redox‐sensitive proteins like IDO1, which are vulnerable to redox‐cycling compounds, and interference of iron chelators with the heme cofactor may lead to promiscuous IDO1 inhibition.^[9]^ Moreover, the temperature and reducing agents during pre‐incubation of IDO1 with compounds can influence potency.[^9a^, ^10^] Even more important, the validation of several IDO1 inhibitors from in vitro to in cellulo is challenging.^[9b]^ These problems might be overcome by means of cell‐based assays in which the protein remains in its native environment. Currently employed cell‐based assays monitoring IDO1 activity utilize *p*‐dimethylamino benzaldehyde (*p*‐DMAB),^[11]^ high‐performance liquid chromatography (HPLC),^[12]^ NFK Green™ ^[13]^ or cucurbit[8]uril‐dimethyldiazaper‐opyrenium dication complex (MP⊂CB[8]).^[14]^ HPLC‐based techniques or *p*‐DMAB require a transfer of cell supernatant^[11]^ which is technologically challenging in screening assays. NFK Green™ displays a low dynamic detection range in cells^[13]^ and MP⊂CB[8]‐mediated detection of Trp requires homogenous diffusion across cell membranes.^[14]^ Thus, there is a high demand for new cell‐based screening assays for identification of IDO1 inhibitor classes.

Here, we describe the discovery of highly potent IDO1 inhibitor chemotypes along with the finding of indirect IDO1 modulators by means of a newly developed cellular assay. The identified compound classes display diverse mechanisms of action. They include modulators of IDO1 expression in cells, inhibitors that interfere with the synthesis of the heme cofactor and compounds that inhibit IDO1 directly, for example, by an interaction with apo‐IDO1.

To determine IDO1‐mediated Kyn production in cells by means of a fluorescence readout, we employed the previously developed coumarin‐based Kyn sensor **2** ^[15]^ (Figure 1 A). In aqueous buffer, aldehyde **2** reversibly reacts with the aniline moiety of Kyn to yield adduct **3** (Figure 1 A).^[15]^ As **2** has not yet been employed in enzymatic or cell‐based assays, we analyzed the spectral properties of **2** and **3** in cell culture medium in the presence of 10 % fetal bovine serum (FBS). **2** absorbs light below 500 nm in cell culture medium and an additional absorbance peak between 525 nm and 560 nm was observed in the presence of Kyn for Schiff base **3** (Figure 1 B). Using ex/em 555/600 nm, the sensor detected different Kyn levels in cell culture medium (Figure 1 C, Figure S1). For further validation, we analyzed the production of Kyn in the human pancreas adenocarcinoma cell line BxPC3. Whereas IDO1 protein is not expressed in BxPC3 cells, stimulation with IFNγ induced IDO1 expression after 24 h, and 48 h were required for sufficient Kyn production (Figure 1 D and Figure S1). For assay optimization, the concentration of the substrate Trp, the incubation time and the sensor concentration were varied (Figure S1), and the IDO1 inhibitors epacadostat (**4**) and BMS‐986205 (**5**) were employed as controls (Figure 1 E).[^10b^, ^11^, ^16^] BxPC3 cells were stimulated with IFNγ for 48 h prior to detection of Kyn by means of sensor **2**. Similar to Kyn detection reagent *p*‐DMAB,^[11]^ **2** detected a dose‐dependent decrease in Kyn levels in BxPC3 cells by epacadostat with a half‐maximal inhibitory concentration (IC_50_) of 198±9 nm, which is in good agreement with the IC_50_ value obtained using *p*‐DMAB (IC_50_=196±4 nm) (Figure 1 F). Automatization and miniaturization to 384‐well format successfully detected the concentration‐dependent decrease in Kyn levels for both IDO1 inhibitors (IC_50_=102±0.1 nm (**4**) and IC_50_=14±0.1 nm (**5**)) (Figure 1 G) with robust assay characteristics (Z′‐factor: 0.76; signal‐to‐background ratio (S/B): 14). For the initial screening, the assay was further miniaturized to 1536‐well format (Z′‐factor: 0.53 and S/B: 14.3). Thus, we developed a robust cellular Kyn assay with a high fluorescence dynamic range that is applicable for high‐throughput analysis.

**Figure 1:**
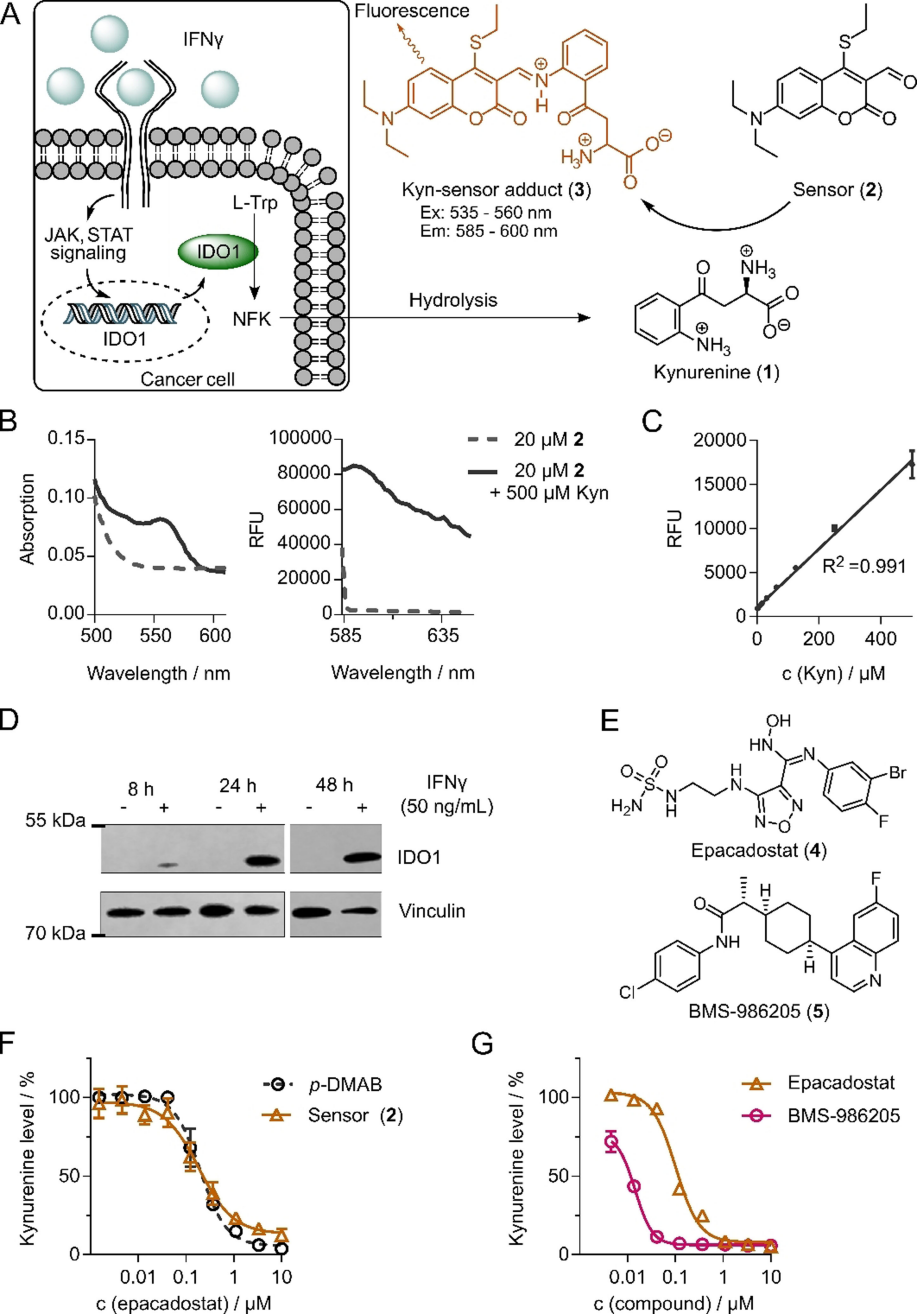
Validation of a coumarin‐based sensor for detection of cellular kynurenine (**1**, Kyn) levels. A) Cellular Kyn assay. Cells are treated with IFNγ, Trp, and compounds. After 48 h, Kyn levels are detected using the Kyn sensor (**2**). B) Absorbance (left) and fluorescence (right) scan of **2** in the absence or presence of Kyn in cell culture medium. C) Detection of **3** by means of fluorescence (ex: 555 nm, em: 600 nm) in the presence of Kyn (0–500 μm) in cell culture medium. D) Induction of IDO1 expression in BxPC3 cells with IFNγ for 8, 24, and 48 h. Representative immunoblots for IDO1 and vinculin as a loading control. See Figure S2 for uncropped blots. E) Chemical structures of the IDO1 inhibitors epacadostat (**4**) and BMS‐986205 (**5**). F) Kyn assay of epacadostat utilizing *p*‐DMAB or sensor **2** for Kyn‐level detection. Data are mean values±S.D., *n*=3. G) Automated Kyn assay for **4** or **5**. Kyn levels were detected using **2**.

Screening of a compound library of 157 332 chemically diverse, commercially available and in‐house synthesized compounds at 7.1 μm using the Kyn assay resulted in a hit rate of 0.62 % (threshold: >50 % inhibition). Compounds that reduced cell viability by more than 25 % and small molecules with pan‐assay interference (PAINS)^[17]^ features were excluded from further analysis (see Supporting Information). Compounds that reduce Kyn production by 70 % or more were subjected to dose‐response measurements. Kyn levels are mainly regulated by IDO1 enzymatic activity in tumor cells.^[18]^ Hit compounds with IC_50_ values <5 μm were analyzed for direct modulation of IDO1 activity which revealed that only 3.9 % of the compounds inhibited IDO1 activity (IC_50_≥0.33 μm), including several chemotypes that have not been linked to IDO1 inhibition before (Figure 2 A, Figure S3 and S4). Hits that did not directly inhibit IDO1 were considered indirect inhibitors (Table 1), including two groups of compounds with annotated mechanism for reduction of Kyn levels. Group 1 downregulates IDO1 expression in cells, whereas group 2 inhibits the synthesis of the IDO1 cofactor heme (Table 1). For example, JAK1/2 inhibitors like ruxolitinib potently reduced Kyn levels (Table 1) by downregulating IDO1 expression (Figure S5), which is in agreement with reduced Kyn levels in cells upon targeting the IFNγ‐induced JAK/STAT pathway.^[4b]^ Furthermore, bromodomain‐containing protein 4 (BRD4) is involved in the epigenetic regulation of *IDO1*.^[19]^ In line with this, BRD4 inhibitors, such as PFI‐1, decreased Kyn levels in cells (Table 1). The second group was comprised of small molecules like succinylacetone that interfere with cellular heme synthesis, reduce the abundance of the cofactor and thereby impair IDO1 activity (Table 1).^[20]^ Thus, the cellular Kyn assay successfully detects direct IDO1 inhibitors and can also uncover modulators of cellular Kyn production with different mechanisms of action.

**Figure 2:**
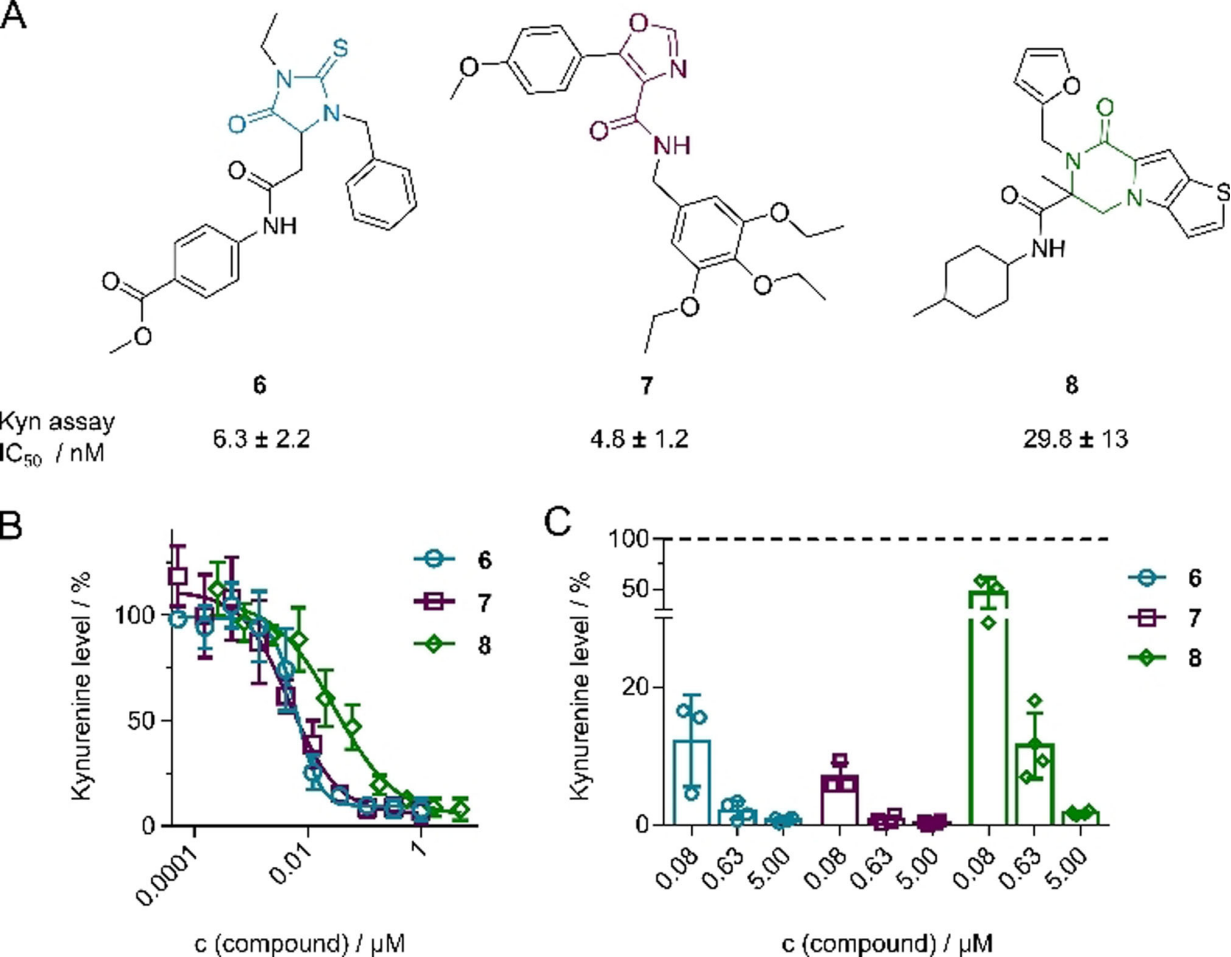
Decrease of cellular Kyn production by identified hit compounds. A) Structures of compound **6**, **7**, and **8** and IC_50_±S.D. values in Kyn assay. B) Kyn assay using **2** in BxPC3 cells treated with **6**, **7** or **8** for 48 h. C) Detection of Kyn levels using LC‐MS/MS. Cells were treated as described in (B). Data are mean values±S.D., *n*≥3.

**Table 1:** Identified compounds with known targets that reduce Kyn levels in the automated Kyn assay. Cell count was evaluated using Hoechst 33342 for compound's cytotoxicity. Data are mean values±S.D., *n*≥3.

Compound	Kyn Assay IC_50_ [μm]	Cell Count IC_50_ [μm]	Annotated Activity	Mode of Action
Momelotinib (CYT387)	0.29±0.06	inactive	ATP‐competitive inhibitor of Janus kinases JAK1 and JAK2	JAK kinase inhibitors
Tofacitinib	0.59±0.008	inactive	Irreversible inhibitor of Janus kinases JAK1 and JAK3	
Ruxolitinib	0.08±0.005	inactive	ATP‐competitive inhibitor of Janus kinases JAK1 and JAK2	
Tofacitinib citrate	0.28±0.03	inactive	Irreversible inhibitor of Janus kinases JAK1 and JAK3	
CEP‐33779	0.45±0.04	inactive	ATP‐competitive inhibitor of Janus kinase JAK2	
GSK1324726A	0.01±0.005	>2	Inhibitor of bromodomain and extra‐terminal (BET) family proteins BRD2, BRD3, and BRD4.	BRD/BET inhibitors
(+)‐JQ1	0.03±0.004	>2	Inhibitor of BRD4 (1/2).	
OTX015	0.03±0.01	>2	Inhibitor of BRD2, BRD3, and BRD4	
I‐BET151	0.07±0.002	inactive	Pan BET family inhibitor	
MZ1	0.09±0.04	1.24	Proteolysis‐targeting chimera (PROTAC) based on JQ‐1. Induces proteasomal degradation of BRD4).	
PFI‐1	0.51±0.06	inactive	Inhibitor of BRD4	
I‐BRD9	>10	inactive	Selective cellular chemical probe for BRD9	
Succinylacetone	0.41±0.22	inactive	Irreversible inhibitor of δ‐aminolevulinic acid dehydratase, which produces porphobilinogen, the precursor of heme.	Heme biosynthesis inhibitors
N‐methyl protoporphyrin IX	2.61±1.64	inactive	Inhibitor of protoporphyrin IX ferrochelatase, which is involved in heme biosynthesis.	

Thiohydantoin inhibitor **6** (IC_50_=6.3±2.2 nm), oxazole‐4‐carboxamide inhibitor **7** (IC_50_=4.8±1.2 nm) and piperazin‐2‐one inhibitor **8** (IC_50_=29.8±13 nm; Figure 2 A and B) are the most potent compounds identified in the screen and were selected for further characterization (see Figure S4 for more derivatives). An orthogonal, sensor‐free method using LC‐MS/MS for Kyn detection validated the reduction of Kyn levels upon treatment of BxPC3 cells with **6**, **7** or **8** (Figure 2 C). In addition, the dose‐dependent reduction of Kyn levels by **6**, **7** and **8** was detected using *p*‐DMAB in BxPC3 cells and in the human ovarian carcinoma cell line SKOV3 upon stimulation with IFNγ as well as in HEK293T cells that transiently express human IDO1 (Figure S6).

To identify potential direct modulators of IDO1, compounds **6**, **7** or **8** (30 μm) were incubated with recombinant human IDO1 protein (rhIDO1) prior to the determination of IDO1 activity. Preincubation of IDO1 with the compounds at 37 °C but not at 20 °C substantially decreased IDO1 activity (Figure 3 A) with IC_50_ values of 0.97±0.59 μm (**6**), 1.52±1.09 μm (**7**) and 5.28±0.92 μm (**8**) (Figure 3 B). A similar observation has been made for the heme‐competitive IDO1 inhibitor BMS‐986205. In cells, heme‐competitive IDO1 inhibitors bind to apo‐IDO1 and prevent heme binding, holo‐IDO1 formation and IDO1 activity.^[10]^ In vitro, holo‐IDO1 is used to evaluate IDO1 activity, and inhibitors binding to apo‐IDO1 require heme dissociation from holo‐IDO1, which in turn requires temperatures of 30 °C or higher.^[10b]^ At lower temperatures, heme‐competitive inhibitors fail to suppress IDO1 activity. However, even at appropriate temperatures, heme dissociation and, thus, formation of apo‐IDO1 in vitro is a reversible and slow process.^[10]^ In vitro, the delay of heme dissociation results in a remaining fraction of active holo‐IDO1 even in the presence of heme‐competitive inhibitors, which reduces the potency of those inhibitors and explains the different IC_50_ values for **6**, **7** and **8** in the enzymatic assay compared to the cell‐based assay.

**Figure 3:**
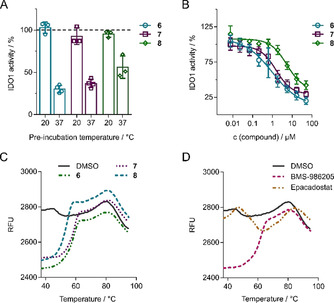
IDO1 is the target of compound **6**, **7**, and **8**. A) Influence of the pre‐incubation temperature on IDO1 activity. IDO1 was incubated with **6**, **7** or **8** at 20 °C or 37 °C for 30 min prior to detection of Kyn levels using **2**. B) IDO1 enzymatic assay. IDO1 was pre‐incubated with the compounds at 37 °C for 40 min prior to the detection of Kyn levels using *p*‐DMAB. Data are mean values±S.D., *n*≥3. C and D) Influence on the melting behavior of IDO1. IDO1 was preincubated with 30 μm of **6**, **7** or **8** (C) or 30 μm BMS‐986205 or epacadostat (D) at 37 °C for 30 min prior to SYPRO orange addition. Representative melting curves of IDO1 are shown (*n*=3, see also Figure S6).

For further characterization, we employed differential scanning fluorimetry (DSF) to explore the melting behavior of IDO1 in the presence of the compounds. Compound **6**, **7** and **8** caused a distinct change of the melting curve compared to the DMSO control (Figure 3 C), and BMS‐986205 induced a similar alteration (Figure 3 D). In contrast, epacadostat, which is a Trp‐competitive IDO1 inhibitor,^[9a]^ led to thermal stabilization of IDO1 with a shift in melting temperature of Δ*T*_m_=4.1±1.2 °C without major change in the melting curve (Figure 3 D and S7). To explore whether heme displacement is responsible for IDO1 inhibition by **6**, **7** and **8**, the spectroscopic properties of IDO1 were analysed in the presence of the compounds. Heme‐containing proteins exhibit a characteristic absorbance peak at 405 nm, the so called Soret band, which corresponds to the electronic state of the iron.^[21]^ A shifted Soret peak indicates a protein‐ligand interaction, and a reduced intensity can reveal heme loss. The IDO1 absorption spectrum in the presence of **6**, **7** or **8** unveiled a clear, concentration‐depended reduction in Soret band intensities similar to the known heme competitor (Figure 4 A). In addition, 14 μm hemin reduced the potency of **6**, **7** and **8** to inhibit rhIDO1 activity as detected by an at least 6.5‐fold increase in the IC_50_ values to 8.2±1.4 μm (**6**), 9.7±0.5 μm (**7**), >30 μm (**8**) (Figure 4 B). These findings strongly suggest that compound **6**, **7** and **8** reduce cellular Kyn levels by competing with heme for apo‐IDO1, thereby suppressing IDO1 activity.

**Figure 4:**
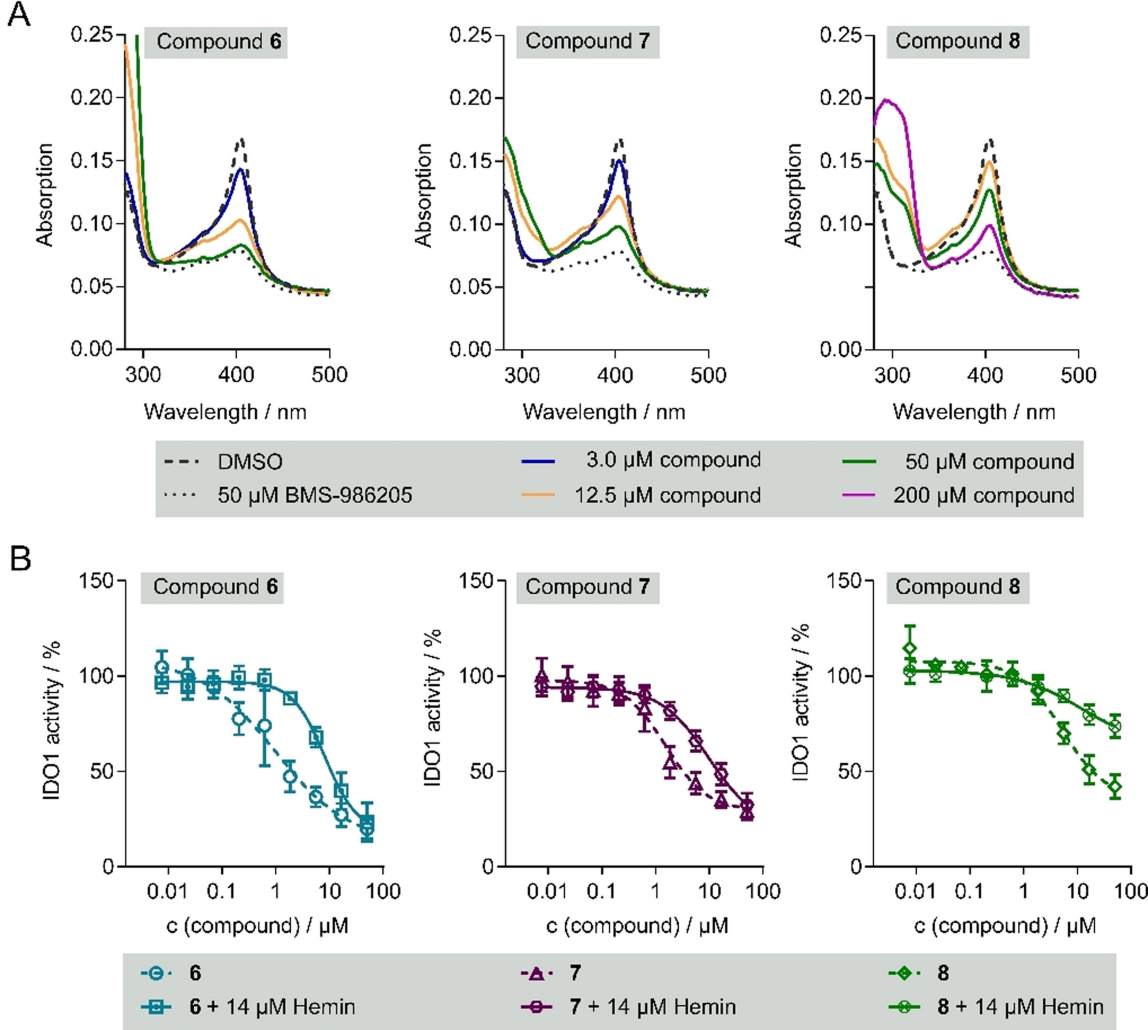
Hit compounds are heme‐competitive IDO1 inhibitors. A) UV/Vis spectrum of IDO1. Human IDO1 was preincubated with the compounds at 37 °C for 120 min prior detection of the UV/Vis spectrum. Representative spectrum (*n*=3). B) Detection of heme competition. Human IDO1 was preincubated with the compounds or the compounds and 14 μm hemin at 37 °C for 40 min prior to detection of Kyn levels using *p*‐DMAB. All data are mean values±S.D., *n*≥3.

In conclusion, we have developed a novel cell‐based method to identify IDO1 inhibitors by fluorescence‐based monitoring of cellular Kyn levels. The method enables the identification of direct IDO1 inhibitors as well as modulators of IDO1 expression or heme biosynthesis, which are considered indirect IDO1 inhibitors. Three IDO1‐inhibitor chemotypes were identified that reduce IDO1 activity by a heme‐competitive mechanism. Our findings underscore the power of a cell‐based approach to identify IDO1‐ and Kyn level modulators and to overcome limitations related to biochemical and biophysical assays employing redox‐sensitive proteins like IDO1.

## Conflict of interest

The authors declare no conflict of interest.

## Supporting information

As a service to our authors and readers, this journal provides supporting information supplied by the authors. Such materials are peer reviewed and may be re‐organized for online delivery, but are not copy‐edited or typeset. Technical support issues arising from supporting information (other than missing files) should be addressed to the authors.

Supplementary
