# Supplementary material for: Cell‐Based Identification of New IDO1 Modulator Chemotypes
Source: Angew Chem Int Ed Engl. 2021 Mar 24;60(18):9869–74. doi: 10.1002/anie.202016004 (PMC8252559; doi:10.1002/anie.202016004)
Supplement: Supplementary file 1 — Supplementary [file ANIE-60-9869-s001.pdf]

Angewandte Chemie
Eine Zeitschrift der Gesellschaft Deutscher Chemiker

Supporting Information

Cell-Based Identification of New IDO1 Modulator Chemotypes

*Elisabeth Hennes, Philipp Lampe, Lara Dötsch, Nora Bruning, Lisa-Marie Pulvermacher, Sonja Sievers, Slava Ziegler, and Herbert Waldmann**

anie_202016004_sm_miscellaneous_information.pdf

Supporting Information

Table of Contents

Supplementary Figures 2
Online Methods 11
Material and Methods 11
Chemical Synthesis Methods 16
Supplementary References 17
Author contributions 17

Supplementary Figures

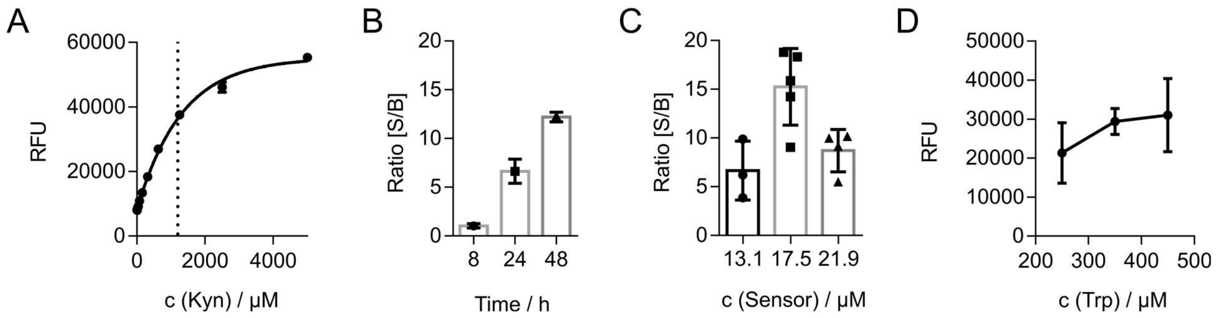

Figure S1: Titration analysis to determine the optimal conditions for the Kyn assay. Related to Figure 1. (A) Kyn titration for determination of the linear detection range of sensor **2**. Detection of fluorescence (ex: 555 nm, em: 600 nm) of **3** in the presence of different Kyn concentrations (0-5000 μM) in cell culture medium. (B) Time course of cellular Kyn production. The Kyn assay was performed in BxPC3 cells upon stimulation with IFNg for 8, 24 and 48 h prior to detection of Kyn levels using LC-MS/MS. Data are mean values ± SD, n=3. (C) Titration of the sensor. The Kyn assay was performed in BxPC3 cells treated with IFNγ for 48 h prior to detection of Kyn levels using different sensor concentrations. Data are mean values ± SD, n ≥ 1. (D) Titration of Trp. The Kyn assay was performed in BxPC3 cells treated with IFNγ and different Trp concentrations for 48 h prior to detection of Kyn levels using 17.5 μM sensor. Data are mean values ± SD, n=3. S/B: signal to background.

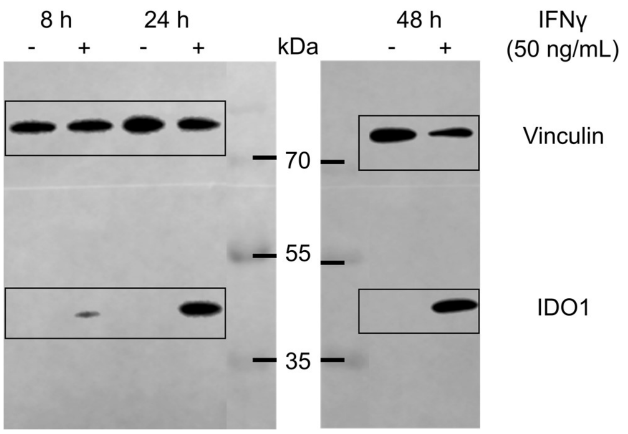

Figure S2: Uncropped immunoblots. Related to Figure 1D.

A

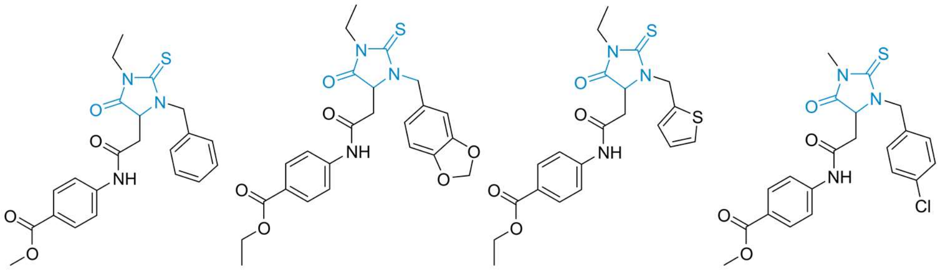

Compound	6	9	10	11
Kyn assay IC_{50} / μM	0.006 ± 0.003	0.008 ± 0.005	0.013 ± 0.01	0.23 ± 0.06
IDO1 activity assay IC_{50} / μM	0.44 ± 0.18	0.62 ± 0.48	0.55 ± 0.23	4.43 ± 1.13
Cell count IC_{50} / μM	inactive	inactive	inactive	inactive

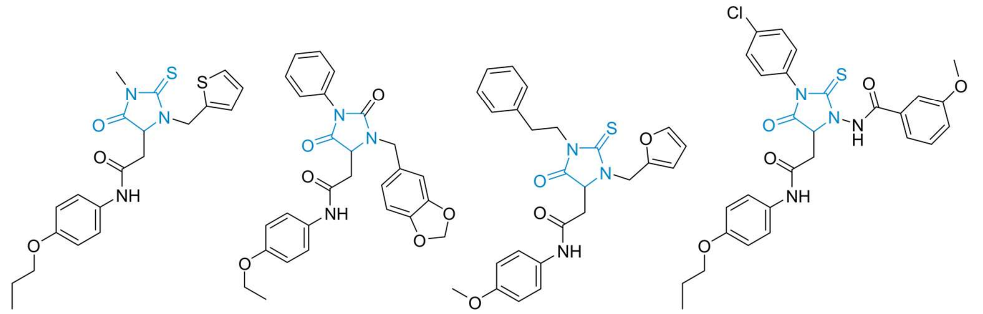

Compound	12	13	14	15
Kyn assay IC_{50} / μM	1.08 ± 0.003	1.11 ± 0.22	≥ 10	inactive
IDO1 activity assay IC_{50} / μM	≥ 30	8.39 ± 1.71	inactive	inactive
Cell count IC_{50} / μM	inactive	inactive	inactive	inactive

B

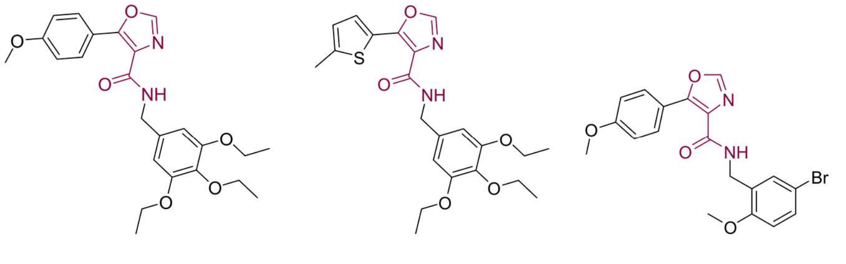

Compound	7	16	17
Kyn assay IC_{50} / μM	0.005 ± 0.001	0.008 ± 0.002	1.01 ± 0.20
IDO1 activity assay IC_{50} / μM	0.73 ± 0.45	1.45 ± 0.39	inactive
Cell count IC_{50} / μM	inactive	inactive	inactive

C

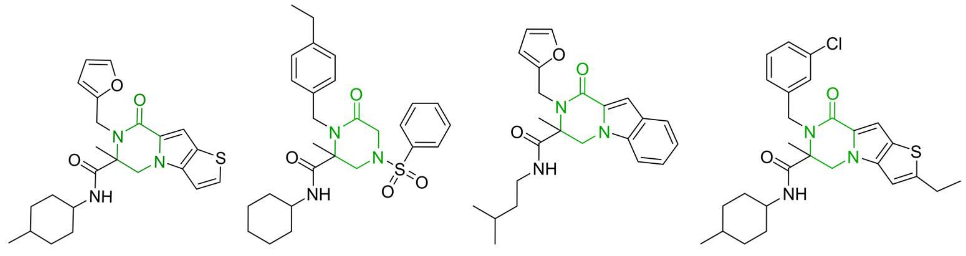

Compound	**8**	**18**	**19**	**20**
Kyn assay IC_{50} / µM	0.05 ± 0.02	0.09 ± 0.03	0.30 ± 0.11	0.40 ± 0.23
IDO1 activity assay IC_{50} / µM	2.35 ± 1.83	1.85 ± 1.06	≥ 60	≥ 60
Cell count IC_{50} / µM	inactive	inactive	inactive	inactive

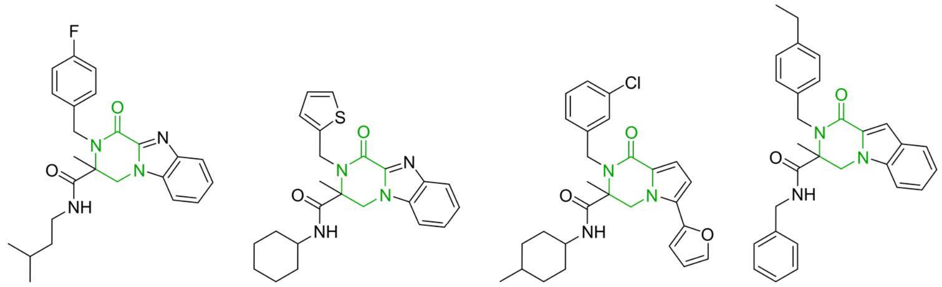

Compound	**21**	**22**	**23**	**24**
Kyn assay IC_{50} / µM	0.44 ± 0.22	0.53 ± 0.07	0.90 ± 0.37	2.60 ± 0.48
IDO1 activity assay IC_{50} / µM	≥ 30	≥ 30	≥ 30	inactiv
Cell count IC_{50} / µM	inactive	inactive	inactive	inactive

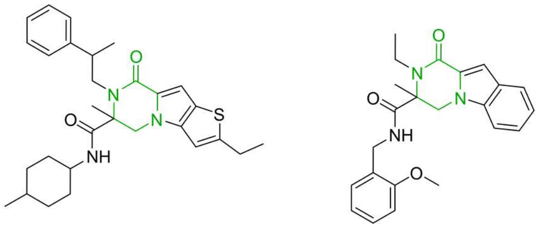

Compound	**25**	**26**
Kyn assay IC_{50} / µM	≥ 10	inactive
IDO1 activity assay IC_{50} / µM	inactive	inactive
Cell count IC_{50} / µM	inactive	inactive

Figure S3: Further derivatives of IDO1 inhibitors 6, 7 and 8. Related to Figure 2A. Kyn was detected utilizing sensor **2** in the Kyn assay as well as in the IDO1 activity assay. (A) Structures and IC_{50} values of derivatives of compound **6**. (B) Structures and IC_{50} values of derivatives of compound **7**. (C) Structures and IC_{50} values of derivatives of compound **8**. IC_{50} values in the automated Kyn assay were determined in BxPC3 cells. IC_{50} values in the IDO1 activity assay were determined with recombinant human IDO1 protein. Cell count was evaluated using Hoechst 33342 for compound cytotoxicity. Data are mean values ± SD, n≥3.

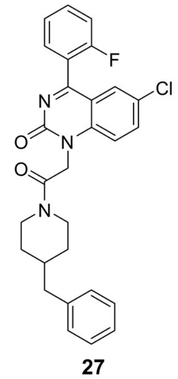 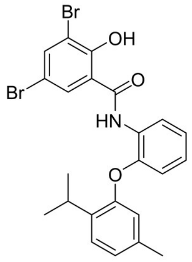 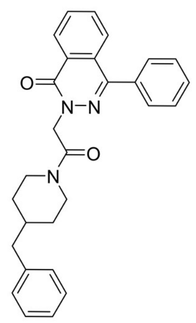 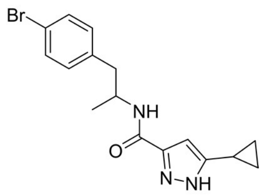

Compound	**27**	**28**	**29**	**30**
Kyn assay IC_{50} / μM	0.08 ± 0.01	0.08 ± 0.001	0.10 ± 0.02	0.35 ± 0.06
IDO1 activity assay IC_{50} / μM	0.33 ± 0.02	0.36 ± 0.02	1.88 ± 0.32	8.91± 1.32
Cell count IC_{50} / μM	inactive	inactive	inactive	inactive

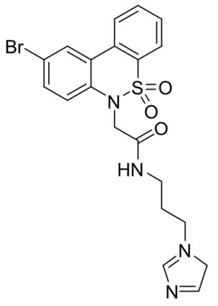 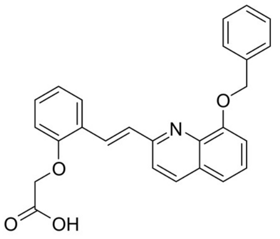

Compound	**31**	**32**
Kyn assay IC_{50} / μM	1.30 ± 0.22	2.00 ± 0.14
IDO1 activity assay IC_{50} / μM	9.91 ± 1.00	6.89 ± 1.99
Cell count IC_{50} / μM	inactive	inactive

Figure S4: Direct IDO1 inhibitors identified in the screen. (A) Structures and IC_{50} values of IDO1 inhibitors identified in the HTS. IC_{50} values in the Kyn assay were determined in BxPC3 cells. Cell count was evaluated using Hoechst 33342 for compound cytotoxicity. IC_{50} values in the IDO1 activity assay were determined with recombinant human IDO1 protein. Data are mean values ± SD, n≥3.

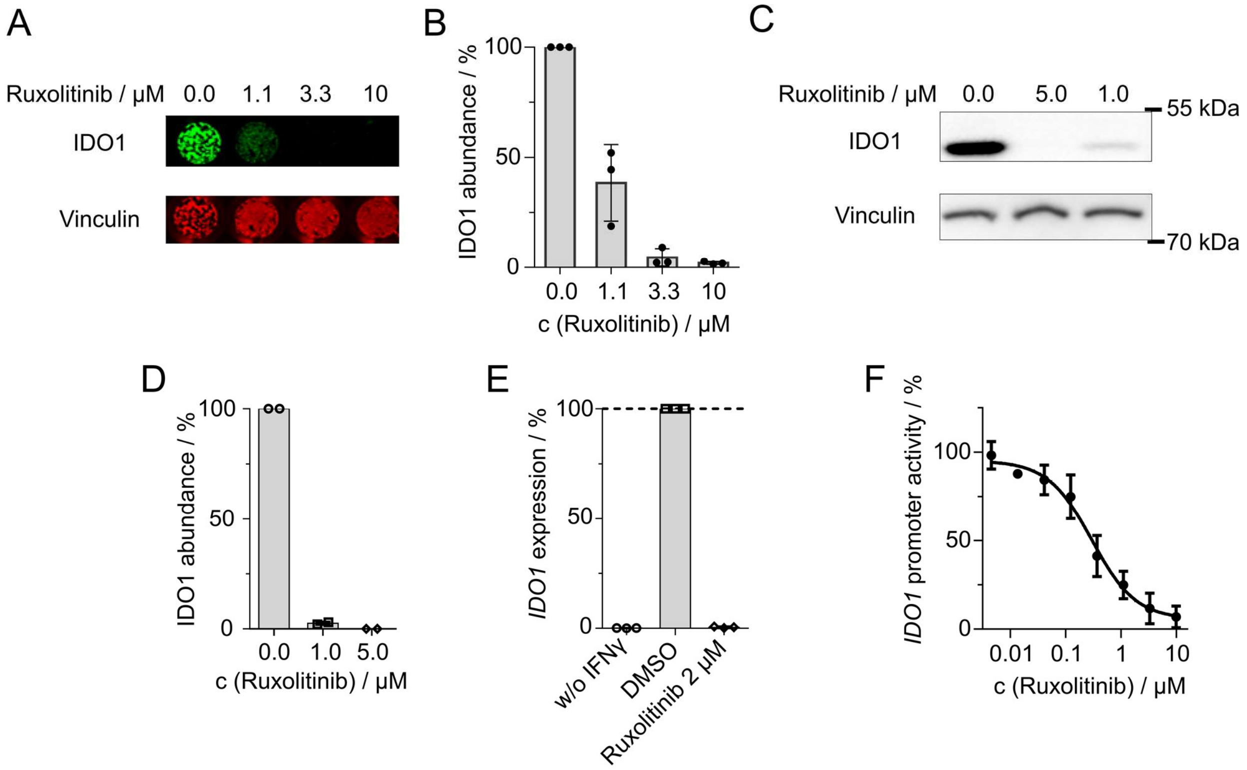

Figure S5: Ruxolitinib, a JAK kinase inhibitor downregulates IDO1 expression and thus reduces Kyn levels. Related to Table 1. (A) In-Cell Western of IDO1 expression in BxPC3 cells upon stimulation with IFNγ and ruxolitinib treatment at indicated concentrations for 48 h. Representative images for IDO1 and vinculin as a loading control. (B) Normalized data of IDO1 abundance from(A). Data are mean values ± SD, n=3. (C) Analysis of IDO1 expression in BxPC3 cells upon stimulation with IFNγ and ruxolitinib treatment at indicated concentrations for 24 h. Representative immunoblots for IDO1 and vinculin as a loading control. (D) Normalized data of IDO1 abundance of the immunoblots from C. Data are mean values ± SD, n=2. (E) mRNA expression level of *IDO1* in BxPC3 cells treated with IFNγ and 2 µM ruxolitinib or DMSO for 24 h. Expression levels are determined using RT-qPCR. (F) *IDO1* promoter reporter gene assay in HEK293T cells transiently transfected with a firefly luciferase construct under the control of *IDO1* promoter and a plasmid for constitutive *Renilla* luciferase expression upon treatment with IFNγ and ruxolitinib or DMSO for 24 h. Data are mean values ± SD, n=3.

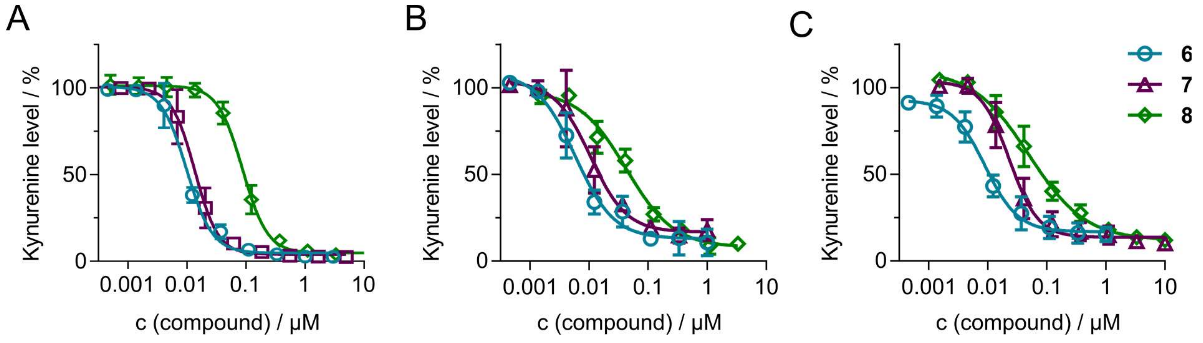

Figure S6: Compounds 6, 7 and 8 inhibit cellular Kyn production. Related to Figure 2B. (A) Kyn assay in BxPC3 cells (A) and SKOV3 cells (B) treated with IFNγ for 48 h prior to detection of Kyn levels using *p*-DMAB. (C) Kyn assay in HEK293T cells that transiently express human IDO1. Compound addition was performed 24 h prior to Kyn levels determination using *p*-DMAB. Data are mean values ± SD, n=3.

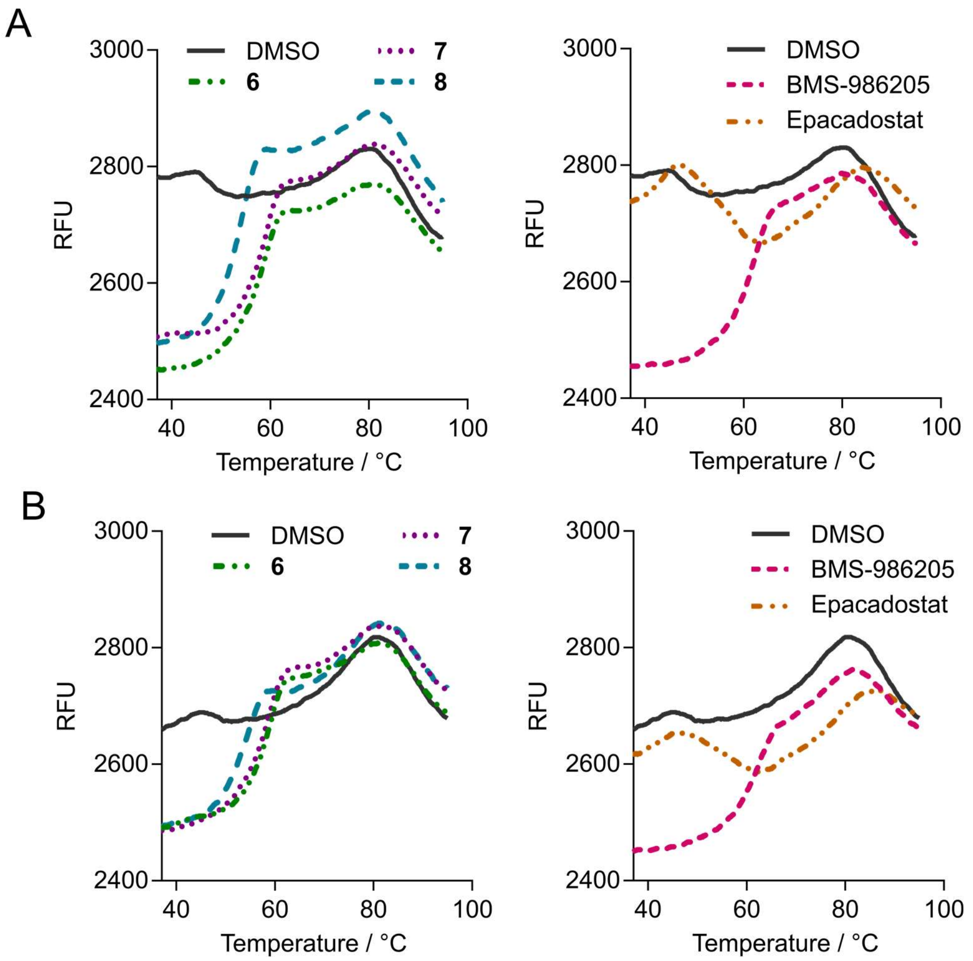

Figure S7: Influence of compounds on the melting behavior of IDO1. Related to Figure 3C and D. Influence of compounds on the melting behavior of IDO1. Human IDO1 was pre-incubated with 30 µM of **6**, **7** or **8** (left) or 30 µM BMS-986205 or epacadostat (right) at 37 °C for 30 min prior to SYPRO orange addition. Replicates 2 (A) and 3 (B) are shown.

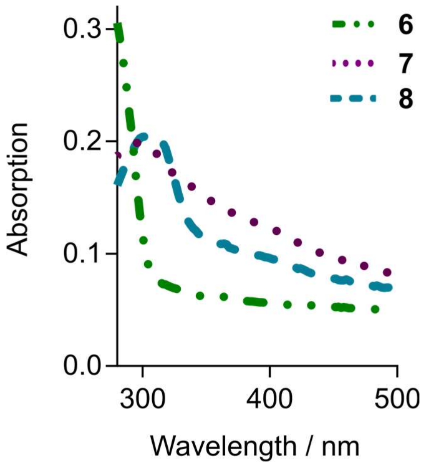

Figure S8: UV-VIS spectrum of compounds 6, 7 and 8. Related to Figure 4A, B and C. Compounds were diluted to 200 µM in 100 mM K-PO_4 buffer and UV-VIS spectrum was monitored.

Online Methods

Material and Methods

Methods

Cell culture

BxPC3 cell line (DSMZ) was grown in RPMI-1640 medium supplemented with 10 % heat inactivated FBS, glucose (final concentration of 4.5 g/l), 2,4 g/l HEPES, 1% L-glutamine and 1% sodium pyruvate. SKOV3 cell line (ATCC) was maintained in McCoy's 5A medium supplemented with 10 % heat inactivated FBS. HEK293T cell line (ATCC) was grown in DMEM supplemented with 10 % FBS and 1 % sodium pyruvate. All cell lines were cultivated in a humidified incubator at 37 °C and 5 % CO_2. All cultures were routinely tested and found to be free of mycoplasma contamination.

Kyn Sensor titration

Absorbance and fluorescence spectra were recorded at 20 °C using a Spark® multimode microplate reader (Tecan, Austria). L-Kynurenine was diluted in cell culture medium without phenol red and supplemented with 10 % FBS and an equal volume of sensor **2** at the indicated concentrations in assay buffer (50 mM H_3PO_4 and 120 mM NaCl (pH=1)) was added. Absorbance spectrum was measured between 500 and 620 nm in 2 nm increments. Fluorescence was measured by using an excitation wavelength of 555 nm. Emission spectrum was recorded between 585 nm and 650 nm or at 600 nm when L-Kyn was titrated.

Screening library

The employed screening library (157,332 compounds) is composed of proprietary (10 %) as well as commercial (90%) compounds including known bioactive small molecules and natural products. Around 4,000 compounds have annotated bioactivity (2.5% of the whole library): the LOPAC library, Prestwick Chemical library, US drug collection as well as Selleckchem kinase and target-specific libraries as well as further hand-picked reference inhibitors. Traditional screening compounds were purchased from ChemDiv and Enamine. Compounds were chosen with cheminformatic tools by applying relaxed criteria for drug-likeness and compliance to Ro5. Compounds **6**, **7** and **8** were purchased from ChemDiv (ID: 5593-1162 (**6**); ID: G528-0384 (**7**); ID: E021-0159 (**8**))

Automated cell-based Kyn assay

High-throughput screening of Kyn pathway modulators was performed in phenol red-free medium supplemented with 10 % FBS in black multiwell plates. BxPC3 cells (1,000 cells/well in 2.5 µl) were seeded in 1536-well plates (Greiner #782086) 24 h prior to compound treatment using Multidrop Combi (Thermofisher Scientific). Plates were loaded into a Spinnaker automation system (Thermofisher Scientific) for the transfer of 2.5 nl of compounds by Echo550 dispenser (Labcyte) and the addition of 1 µl of medium containing IFNγ and L-Trp (final concentration of 50 ng/mL and 380 µM respectively). In the control wells, which were included on every screening plate, 1 µl of medium containing L-Trp only was added. After a brief centrifugation step, the plates were incubated for 48 h at 37 °C and 5 % CO_2. Afterwards, TCA was added to a final concentration of 7 % to each well and incubated for 10 min 37 °C prior to a centrifugation step of 10 min at 1620 x g. Detection of Kyn was performed by addition of sensor (**2**) to a final concentration of 17.5 µM in assay buffer. Fluorescence intensity (excitation: 535 nm, emission: 595 nm) was detected using a Spectramax Paradigm reader (Molecular Devices). Data was normalized to DMSO-treated cells. All HTS data was analyzed using the Quattro Software Suite (Quattro Research GmbH).

After the primary screening small molecules with pan-assay interference (PAINS)[1] features were excluded from further analysis. PAINS filtering was done computationally using the structural features in SMARTS encoding form. In addition, structural features described by Dahlin *et al.*[2] were manually transformed into SMARTS and also removed. The PAINS filter was implemented as a Pipeline Pilot protocol that takes a list of compounds as input, filters out PAINS-containing structures by SMARTS-based substructure matching, and outputs only the PAINS-free compounds.[1-3] Afterwards, ca. 2800 hit compounds were picked from the automated compound storage (A3 store, Brooks) and re-screened in triplicate using the Kyn assay and determination of cell count as described below.

Dose-response analyses of hit compounds that reduce Kyn levels were performed in 384-well plates in eight three-fold dilution steps starting from 10 µM. Thereby 98.5 % of the hit compounds that reduce Kyn levels by 70 % or more were confirmed as Kyn level modulators. BxPC3 cells (4000 cells/well) were seeded in 384-well plates (Corning #3770) and treated as described for 1536-well plates. As toxic compounds also reduce Kyn levels, a cell count for the evaluation of cytotoxic compounds was performed prior to TCA addition and Kyn detection. Therefore, 5 ng Hoechst (33342) were added to each well. After incubation for 30 min at 37°C, cells were imaged (excitation: 377/50 nm, emission: 447/60 nm) using the ImageXpress Micro XL device (Molecular Devices) followed by image analysis using MetaXpress and the Cell Proliferation HT module. All measurements were performed at least in triplicates.

Manual cell-based Kyn assay (sensor **2**)

BxPC3 (6,000 cells/well,) were seeded in 384-well plates, respectively, 24 h prior to compound treatment. 50 ng/mL, 380 µM L-Trp and compounds at indicated concentrations were added and incubated for 48 h. Afterwards, trichloroacetic acid was added to a final concentration of 7 % to each well and incubated for

10 min 37 °C followed by centrifugation for 10 min at 1620 x g. Finally, for the detection of Kyn, one volume of 2 % ((w/v)) of Ehrlich reagent in acetic acid was added and fluorescence intensity (excitation: 555 nm, emission: 600 nm) was detected by means of Spark® multimode microplate reader (Tecan, Austria). Kyn levels were determined by subtracting the absorbance value at 650 nm and presented relative to the DMSO control. Dose-response curves and IC_{50} values were generated and fitted with GraphPad Prism 6.0 (GraphPad software, USA) using four-parameter variable slope non-linear regression curve fit.

Manual cell-based Kyn assay (*p*-DMAB)

BxPC3 or SKOV3 cells (2,0000 cells/well or 6,500 cells/well,) were seeded in 96-well or 384-well plates, respectively, 24 h prior to compound treatment. 50 ng/mL (96-well) or 5 ng/mL (384-well) IFNγ, 380 µM L-Trp (96-well) or 450 µM L-Trp (384 well) and compounds at indicated concentrations were added and incubated for 48 h. Afterwards, trichloroacetic acid was added to a final concentration of 7 % to each well and incubated for 10 min 37 °C followed by centrifugation for 10 min at 1620 x g. Finally, for the detection of Kyn, one volume of 2 % ((w/v)) of Ehrlich reagent in acetic acid was added and absorbance was measured at 492 nm and at 650 nm as background control on Spark® multimode microplate plate reader (Tecan, Austria). Kyn levels were determined by subtracting the absorbance value at 650 nm and presented relative to the DMSO control. Dose-response curves and IC_{50} values were generated and fitted with GraphPad Prism 6.0 (GraphPad software, USA) using four-parameter variable slope non-linear regression curve fit.

Label-free Kyn detection

BxPC3 cells were seeded in 96-well plates 24 h prior to compound treatment. 50 ng/mL IFNγ, 380 µM L-Trp and compounds at indicated concentrations were added and incubated for 48h. Afterwards, TCA to a final concentration of 7 % was added to each well and incubated for 10 min 37 °C. Samples were centrifuged for 10 min at 1620 x g followed by the detection of Kyn in solution by HPLC-MS/MS using the LTQ Velos Pro and Dionex HPLC (Thermo Fisher Scientific). Data were analyzed using Thermo Xcalibur™ (Thermo Fisher Scientific) and presented GraphPad Prism 6.0 (GraphPad software, USA).

HEK293T-based Kyn assay

HEK293T cells were reverse transfected with 1 µg pCMV3-IDO1 using Lipofectamine 2000 while seeding in a 96-well plate (25,000 cell/well) 20 h prior to addition of 500 µM L-Trp and compounds at the indicated concentrations. After 24 h of compound incubation, trichloroacetic acid was added to a final concentration of 7 % to each well and incubated for 10 min 37 °C. Samples were centrifuged for 10 min at 1620 x g.

Finally, for the detection of Kyn, one volume of 2 % (w/v) of Ehrlich reagent in acetic acid was added and absorbance at 492 nm and at 650 nm as background control was measured on Spark® multimode microplate plate reader (Tecan, Austria). Kyn levels were determined by subtracting the absorbance value at 650 nm and presented relative to the DMSO control. Dose-response curves and IC_{50} values were generated and fitted with GraphPad Prism 6.0 (GraphPad software, USA) using four-parameter variable slope non-linear regression curve fit.

IDO1 promoter reporter gene assay

$2.5x10^6$ HEK293T cells were reverse transfected with 4 µg pXPG-IDO1-FL (kindly provided from Gina Doody, Leeds[4]) and 0.3 µg pRL *Renilla* luciferase control reporter plasmid using Lipofectamine 2000. Afterwards, cells were seeded in a 96-well plate (25,000 cell/well) and 24 h later treated with 50 ng/mL IFNγ and compounds at the indicated concentrations. After 24 h of compound incubation, cells were lysed and luciferase activities measured using the dual Luciferase Reporter Assay System (Promega, USA) on Spark® multimode microplate plate reader (Tecan, Austria). Firefly luciferase values were normalized to the respective *Renilla* luciferase values. Dose-response curves and IC_{50} values were generated and fitted with GraphPad Prism 6.0 (GraphPad software, USA) using four-parameter variable slope non-linear regression curve fit.

Immunoblotting

For analysis of IDO1 levels, BxPC3 cells were stimulated with 50 ng/mL IFNγ for 8, 24 or 48 h followed by cell lysis utilizing 150 mM sodium chloride in 50 mM Tris (pH 8.0) supplemented with 1% NP-40 alternative, protease and phosphatase inhibitors. Protein concentrations were determined by means of DC protein assay (Bio-Rad) and 1 x SDS loading buffer was added to the lysate prior to protein separation by a 10 % SDS-PAGE. Afterwards, proteins were transferred to a polyvinylidene difluoride (PVDF) membrane using a wet-tank blotting system (Bio-Rad). IDO1 and Vinculin as loading control were detected using the primary antibodies anti-IDO1 (rabbit) and anti-vinculin (mouse) in 5 % milk in tris-buffered saline with 0.05 % (v/v) Tween-20 (TBS-T). Primary antibodies were incubated at 4 °C overnight and detected with horseradish peroxidase-conjugated (Pierce) or IRDye-conjugated secondary antibodies (LI-COR Biosciences) for 1 h at room temperature. Washing steps were performed with TBS-T. Protein bands were visualized by the Odyssey Fc imaging system (LI-COR, USA) using a chemiluminescent or fluorescent readout.

In-Cell Western

For analysis of IDO1 levels, BxPC3 cells were treated with 50 ng/mL IFNγ and the respective compound concentrations or DMSO. After 48 h of incubation, cells were washed with PBS prior to fixation using 3.7 %

paraformaldehyde in PBS for 20 min at room temperature followed by permeabilization using 0.2 % Triton X-100 in PBS for 20 min at room temperature. Afterwards, cells were washed with PBS prior to blocking with 5 % skimmed milk in TBS for 1 h at room temperature. Samples were incubated with rabbit anti-IDO1 antibody (1:1000 in 5 % milk TBS-T) and mouse anti-vinculin antibody (1:5000 in Odyssey Blocking buffer) at 4 °C overnight and detected with IRDye-conjugated secondary antibodies (LI-COR Biosciences) 1:500 in Odyssey Blocking buffer for 1 h at room temperature. Washing steps were performed with TBS-T. Plates were scanned using the Odyssey CLx imaging system (LI-COR, USA). After background subtraction, IDO1 fluorescence was normalized to the fluorescence of vinculin as a control.

Reverse transcription-quantitative PCR (RT-qPCR)

BxPC3 (1 x 10^5 cells/well) were seeded in 12-well plates 24 h prior. 24 h later, cells were treated with 50 ng/mL IFNγ and the respective compound concentrations or DMSO for 24 h. Afterwards, total RNA was purified using RNeasy mini kit (Qiagen) and reverse transcribed with Quantitect Reverse transcriptase (Qiagen) according to the manufacturer's instructions. The obtained cDNA was diluted 5-fold. 3 µL diluted cDNA were employed (in triplicates) as a template for quantitative PCR using QuantiFast SYBR green PCR kit (Bio-Rad) on iQ™5 Real-Time PCR Detection System (Bio-Rad). *IDO1* expression levels were normalized to the levels of *GAPDH* (that was used as a reference gene in each sample). Relative quantification was performed using the $2^{-\Delta\Delta Ct}$ method.[4] Primer sequences: GAPDH forward: 5'-GTCTCCTCTGACTTCAACAGCG-3'; GAPDH reverse: 5'-ACCACCCTGTTGCTGTAGCCAA-3', IDO1 forward: 5'-GCCTGATCTCATAGAGCTTGGC-3', IDO1 reverse: 5'-TGCATCCCAGAACTAGACGTGC-3'.

Biochemical IDO1 assay

1 µM full length human recombinant IDO1 (rhIDO1) protein expressed in *E.coli* [5] was incubated in 50 mM potassium phosphate buffer (pH 6.5) with the respective compounds or DMSO for 40 min at 37 °C prior to addition of 10 mM ascorbic acid, 10 µM methylene blue, 2 mM L-Trp and 100 µg/mL catalase (50 nM rhIDO1 when pre-incubated at 20 °C). For the hemin competition assay, 1 µM rhIDO1 was incubated with 14 µM hemin and the respective compound or DMSO for 40 min at 37 °C prior the addition of the aforementioned components. Upon incubation for 60 min at 37 °C. TCA was added to a final concentration of 7 %. After incubation at 37 °C for 30 min an equal volume of 2 % ((w/v)) of Ehrlich reagent in acetic acid or one volume of 40 µM **2** in assay buffer was added and absorbance (492 nm and at 650 nm as background control) or fluorescence (excitation: 535 nm, emission: 595 nm) was measured. Kyn levels were determined and presented relative to the DMSO control. Dose-response curves and IC_{50} values were generated and fitted with GraphPad Prism 6.0 (GraphPad software, USA) using four-parameter variable slope non-linear regression curve fit.

Differential scanning fluorimetry (DSF)

0.5 mg/mL (11 µM) rhIDO1 were mixed with the respective compound or DMSO in 50 mM potassium phosphate buffer (pH 6.5), 20 mM sodium ascorbate and 5 µM methylene blue. The protein solution was incubated for 30 min at 37 °C prior to addition of SYPRO orange dye (20,000 x, S66650, Thermo Fisher) with a final concentration of 10 x. Fluorescence intensity was measured every 0.2 °C from 37 °C up to 95 °C utilizing the CFX96 Real-Time System. Melting temperatures were calculated using GraphPad Prism 6.0 (GraphPad software, USA).

UV/VIS analysis

10 µM rhIDO1 DMSO in 100 mM potassium phosphate buffer (pH 6.5) were incubated with the indicated compounds or DMSO (2 %) and incubated for 3 hours at 37°C. Afterwards, a centrifugation step for 10 min at 4000 rpm was performed and the absorbance spectrum was measured at room temperature. The UV/VIS spectra were monitored by the Spark® multimode microplate reader (Tecan, Austria) between 280 to 550 nm in 2 nm increments.

Chemical Synthesis Methods

Synthesis of Sensor **2**

Synthesis and characterization of **2** was reported previously by Klockhow and Glass *et al.*[6]

Supplementary References

[1] J. B. Baell, G. A. Holloway, *Journal of Medicinal Chemistry* **2010**, *53*, 2719-2740.
[2] J. L. Dahlin, J. W. Nissink, J. M. Strasser, S. Francis, L. Higgins, H. Zhou, Z. Zhang, M. A. Walters, *J Med Chem* **2015**, *58*, 2091-2113.
[3] S. Saubern, R. Guha, J. B. Baell, *Mol Inform* **2011**, *30*, 847-850.
[4] K. J. Livak, T. D. Schmittgen, *Methods* **2001**, *25*, 402-408.
[5] T. K. Littlejohn, O. Takikawa, D. Skylas, J. F. Jamie, M. J. Walker, R. J. Truscott, *Protein Expr Purif* **2000**, *19*, 22-29.
[6] J. L. Klockow, T. E. Glass, *Organic Letters* **2013**, *15*, 235-237.

Author contributions

E.H. performed the synthesis of **2**, validated and optimized conditions for the screening assay, carried out cellular Kyn assays in BxPC3 and SKOV3 cells, HPLC-MS/MS-based Kyn measures, expressed and purified IDO1 and performed the biochemical studies. P.L. optimized the automated IDO1 enzymatic assay and performed the IDO1 activity assay for all screening hits. N.B. performed the validation of **2** for its applicability, optimized assay conditions and carried out the expression analysis of IDO1 in BxPC3 cells. L.D. performed the Kyn assay in HEK293T cells and the *IDO1*-promoter reporter gene assay. L-M. P. contributed to the DSF experiments. S.S. adapted the assay to high-throughput format and carried out the analysis of high-throughput data. S.Z and H.W. designed the research. E.H., S.Z. and H.W. wrote the paper.
